# Attitudes, attributions, and usage patterns of primary care patients with regard to over-the-counter drugs—a survey in Germany

**DOI:** 10.1007/s10354-022-00967-6

**Published:** 2022-09-23

**Authors:** Julian Wangler, Michael Jansky

**Affiliations:** Centre for General Medicine and Geriatrics, University Medical Center, Johannes Gutenberg University Mainz, Am Pulverturm 13, 55131 Mainz, Germany

**Keywords:** Non-prescription drugs, Over-the-counter drugs, Self-medication, General practitioner, Waiting room survey, Rezeptfreie Medikamente, Over-the-Counter-Medikamente, Selbstmedikation, Hausarzt, Wartezimmerbefragung

## Abstract

Studies show that over-the-counter drugs are widely used by consumers. Moreover, there is a huge selection available and they are prominently featured in advertising. To date, there exist only a few studies that shed light on the attitudes, attributions, and usage patterns of patients with regard to use of over-the-counter drugs. An anonymized explorative waiting room survey was conducted among 900 patients in 60 GP practices in the German states of North Rhine-Westphalia, Hesse, and Rhineland-Palatinate. As well as the descriptive analysis, a *t* test was applied to independent random samples, in order to identify significant differences between two groups. 65% of respondents reported using over-the-counter drugs frequently or occasionally. With regard to effects, risks, and side effects, 54% state that they usually take advice from their GP and/or pharmacist before purchasing or taking over-the-counter preparations. For 56%, the package information leaflet is a frequent source of information about the over-the-counter drugs used. The respondents consider over-the-counter preparations to be particularly suitable for (preventive) treatment of colds, flu symptoms, and pain management. The widespread perception of over-the-counter drugs as simple to use (62%), low-dose (69%), and low-efficacy (73%) products does not always correspond to the actual capabilities and risks of over-the-counter self-medication. Given the easy availability of over-the-counter drugs and their strong presence in advertising, it is important that patients have a realistic idea of the capabilities and risks of over-the-counter products. In addition to the advice provided by pharmacists, the trusting, long-standing support provided by GPs and their ongoing information and advice services play a central role in this. It would be advisable to give more attention to this public health concern and to promote initiatives to make patients more aware of the risks regarding consumption of drugs without medical consultation.

**Supplementary Information:**

The online version of this article (10.1007/s10354-022-00967-6) contains supplementary material, which is available to authorized users.

## Abstract (de)

Studien zeigen, dass rezeptfreie Medikamente („over-the-counter drugs“) von Verbraucher*innen verbreitet genutzt werden. Zudem erfreuen sie sich eines großen Angebots und sind in der Werbung prominent vertreten. Bislang fehlt es an belastbaren Untersuchungen, die beleuchten, welche Einstellungen, Zuschreibungen und Nutzungsmuster bei Patient*innen in Bezug auf den Einsatz von OTC-Medikamenten gegeben sind. Im Zuge einer explorativen Wartezimmerbefragung erfolgte eine anonymisierte Befragung von 900 Patient*innen in 60 hausärztlichen Praxen in den Bundesländern Nordrhein-Westfalen, Hessen und Rheinland-Pfalz. Neben der deskriptiven Analyse kam zur Feststellung von signifikanten Unterschieden zwischen 2 Gruppen ein *t*-Test bei unabhängigen Stichproben zum Einsatz. Rezeptfreie Medikamente werden von 65 % der Befragten häufig oder gelegentlich konsumiert. Mit Blick auf Wirkungen, Risiken und Nebenwirkungen holen 54 % vor dem Kauf bzw. der Einnahme i. d. R. Rat durch Hausärzt*innen und/oder Apotheker*innen ein. Die Packungsbeilage nutzen 56 % häufig als Informationsmittel über die genutzten OTC-Medikamente. OTC-Präparate werden von den Befragten v. a. zur (präventiven) Behandlung von Erkältungsbeschwerden, grippalen Symptomen oder Schmerzbehandlung wahrgenommen. Hierbei werden rezeptfreie Arzneimittel als unkompliziert anzuwendende (62 %), niedrig dosierte (69 %) und schwach wirksame (73 %) Produkte erlebt. Dies korrespondiert nicht immer mit den realen Möglichkeiten und Risiken einer OTC-Selbstmedikation. Die breite Verfügbarkeit von OTC-Medikamenten und ihr Stellenwert in der Werbung machen es erforderlich, dass Patient*innen eine realistische Vorstellung von den Möglichkeiten und Risiken von OTC-Produkten haben. Jenseits der Beratung durch Apotheker*innen kommt es dabei v. a. auf die vertrauensvolle, langjährige Begleitung durch Hausärzt*innen sowie ihre Informations- und Beratungstätigkeit an. Es erscheint ratsam, OTC-Medikation als Thema der öffentlichen Gesundheitskommunikation zu begreifen und Initiativen zu fördern, die das Bewusstsein der Patient*innen für die Risiken eines Medikamentenkonsums ohne ärztliche Konsultation stärken.

## Introduction

In Germany, as in other Western countries, over-the-counter (OTC) drugs have acquired great importance, and, accordingly, the consumption of these over-the-counter drugs has been increasing for years [1–5]. For example, studies have shown that a substantial proportion of the population regularly reaches for over-the-counter analgesics such as paracetamol and ibuprofen, without always considering any potential side effects [2, 6, 7].

In studies, up to 48% of the German citizens surveyed stated that they used over-the-counter drugs for self-medication; in some of these cases, more than three preparations were taken daily [8–10]. The most commonly used OTC group are nonsteroidal anti-inflammatories and other analgesics [7, 11]. In parallel, an increasing consumption of dietary supplements such as vitamins and minerals can be observed [7, 12].

When used properly, over-the-counter drugs can be beneficial [2, 13]. For example, OTC preparations potentially enhance patients’ sense of empowerment, personal responsibility, and sovereignty. In addition, they allow a high degree of independence, since they do not require a mandatory consultation with the doctor or pharmacist. This simplified care chain results in cost and time savings for both patients and the healthcare system [1, 9, 13].

Nevertheless, self-medication may involve considerable risks, especially when different preparations are combined and interact with each other [14–16]. This risk exists, for example, in multimorbid patients who are already receiving basic pain medication [1]. Also, casual use of OTC preparations can result in excessive doses and associated negative impacts [9]. One can imagine, for example, that the “over-the-counter” classification may lead some patients to mistakenly assume that these drugs only have a minimal effect and are therefore not harmful, even if they increase their intake or use them excessively. It is also conceivable that the desire for a rapid treatment outcome leads to maximum dosages or other recommendations contained in the package insert being ignored. It is not uncommon for consumers of OTC drugs to forego medical consultation [1, 6].

### Research interest

To date, there is a lack of studies from (primary) medical care in German-speaking countries that focus on attitudes, attributions of characteristics, and patterns of use among patients with regard to the use of this group of drugs [17–19]. Accordingly, the present study looks at the views, attitudes, and behaviors of primary care patients with regard to OTC drugs.

## Materials and methods

### Study design and recruitment

In order to obtain a picture of the attitudes of primary care patients, it was decided to conduct a written, anonymized waiting room survey.

A total of 900 patients in 60 GP practices in the states of North Rhine-Westphalia, Hesse, and Rhineland-Palatinate were to be anonymously surveyed. Practices were selected according to a set of guiding criteria designed to obtain a mixed sample of GP practices and thus a heterogeneous patient clientele. Among other things, care was taken to give equal weight to single-handed and group practices and to urban and rural practice locations. In addition, emphasis was placed on ensuring a broad geographic distribution.

When contacted, the practice owners were informed of the intent and content of the study by means of an invitation letter; implementation procedures were also explained. If interest was expressed, a preliminary interview was requested with the practice manager and, firstly, official consent to participate in the study was obtained. Secondly, this interview was intended to ensure that the study was similarly conducted at all sites. During the preliminary interview, it was agreed that either the practicing doctor or the practice staff would approach the patients about the possibility of participating in the study. Questionnaires were to be handed out to 15 patients from diverse socioeconomic groups per practice (ideally at the reception by the practice staff); the questionnaires had to be handed in before leaving the practice.

Recruitment took place in the first half of 2021. Ultimately, the targeted 900 questionnaires were received back from 60 practices. The sample of patients interviewed breaks down as follows:

- Gender: 50% male, 50% female
- Average age: 47 years (median: 46 years; min: 18/max: 91)
- Highest educational attainment: 18% primary/secondary modern school, 38% grammar school or similar, 35% technical university/university entrance qualification, 9% other
- 62% employed, 38% unemployed
- Practice location: 50% rural/small town, 50% medium-sized town/city

### Investigation tool

The 14-question questionnaire was developed primarily on the basis of a literature review (including [1, 2, 7, 19, 20]). The tool was enriched by means of preliminary interviews with five GPs; the item sets (questions 9, 11) in particular were developed on this basis. They were also given the sheet for the first stage of the pretest. The questionnaire (completion time approximately 10 min) consisted of four blocks:

- Buying and usage patterns relating to over-the-counter drugs
- Information-seeking behavior regarding over-the-counter drugs
- Behavior of respondents when using the Internet
- Attitudes regarding properties, (side) effects, and preferred areas of application

The second stage of pretesting was done by conducting a pilot study with a small sample.

Sociodemographic characteristics collected were gender, age, German state, highest educational attainment, and occupation.

### Data analysis

The data were analyzed by means of SPSS 23.0 (IBM Corp., Armonk, NY, USA). As well as the descriptive analysis, a *t* test was applied to independent random samples in order to identify significant differences between two groups (mean difference at the level *p* < 0.001).

## Results

### Acquisition and use of over-the-counter drugs

Overall, 65% of respondents report using OTC drugs frequently or occasionally (35% rarely). The local pharmacy is the most popular place for purchasing over-the-counter drugs; 28% of respondents occasionally obtain OTC preparations via the Internet, these orders primarily being placed via online pharmacies.

First, there are noticeable differences between the sexes. For example, 42% of women report purchasing OTC drugs frequently, as compared to only 19% of men (*p* < 0.001). Secondly, 43% of individuals with a higher level of formal education report purchasing OTC products frequently; this is only 26% (*p* < 0.001) among individuals with lower educational attainment.

Whereas 46% of respondents state that they do not usually seek advice or obtain information about the effects, risks, or side effects of OTC preparations before buying or taking them, 54% state that they usually do seek advice from their GP and/or pharmacist. In addition, for 56%, the package information leaflet is a frequent source of information about the over-the-counter drugs they use. Women seek advice from doctors or pharmacists more often than men (66% vs. 44%, *p* < 0.001). They also place greater value on consulting the package information leaflet (73% vs. 41%, *p* < 0.001).

### Areas of application for over-the-counter drugs

**Fig. 1:**
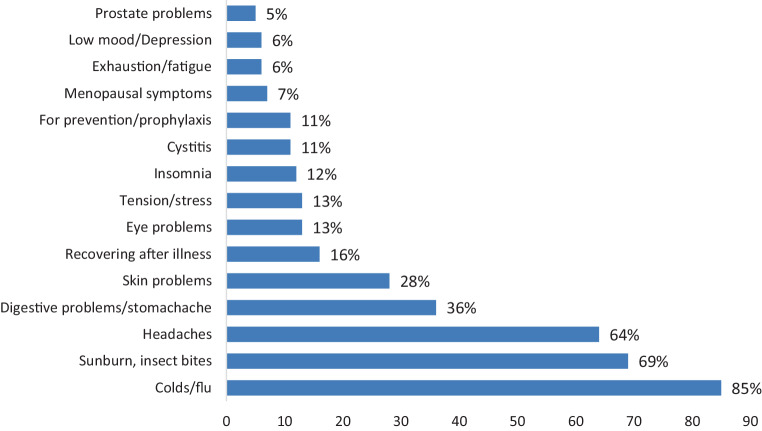
Suitability of over-the-counter drugs according to area of application (*N* = 900). Question: *In your opinion or experience, for which complaints do you think over-the-counter drugs are particularly suitable?*

In the opinion or experience of well over 80% of respondents, OTC drugs are particularly suitable for treatment of colds and flu symptoms, followed by the treatment of sunburn, insect bites, digestive problems, or headaches (Fig. 1).

Compared to 2% of women, 21% of men report that OTC drugs are good for prophylaxis (*p* < 0.001). Similarly, compared with women, men are significantly more likely to say over-the-counter drugs are useful for building yourself up after an illness (29% vs. 4%, *p* < 0.001).

### Perception of over-the-counter drugs

Many respondents associate the existence of over-the-counter medicines primarily with a reduction in the financial burden on health insurers (Table 1). This fact is attributed in part to insufficiently proven efficacy.

**Table 1:** Assumptions about over-the-counter drugs (*N* = 900). Question: *Here are several assumptions about over-the-counter drugs. Which statements do you think are true and which are not?*

Over-the-counter drugs	Mostly true (in %)	Mostly untrue (in %)	Not specified (in %)
… are there to relieve the financial burden on health insurers, because patients have to pay for such drugs themselves	74	23	3
… have fewer side effects than prescription drugs	39	56	5
… are not paid for by health insurers, because there is no clear evidence of their efficacy	22	74	4
… are much easier to use and take than prescription drugs	35	62	3
… are in lower doses than prescription drugs	69	29	2
… save me from having to go to the doctor for every minor complaint	64	32	4
… are freely available to buy, because they have been on the market for a long time and are therefore well tried and tested	45	51	4

Although more than half of respondents doubt that non-prescription equates to fewer side effects, other responses indicate that OTC preparations are more likely to be associated with treating minor ailments and self-management of illnesses. In addition, almost every second person has the idea that over-the-counter drugs are freely available because they have been tested for a long time and their effects and side effects are therefore well known and can be calculated.

In the sample, 52% of men report that OTC drugs have fewer side effects than prescription drugs, as opposed to 26% of women (*p* < 0.001). Men are also much more likely than women to assume that OTC drugs are significantly easier to take (49% vs. 21%, *p* < 0.001).

For around one in two, OTC drugs can readily be used as required (Table 2). Around two-thirds of patients believe that OTC drugs can cause harm if taken incorrectly and should not be taken on a long-term basis. At the same time, OTC preparations are considered harmless by a considerable percentage of respondents and are associated with minimal side effects. A similar number of respondents believe that OTC drugs have a performance-enhancing effect or aid recovery after an illness. Three out of four respondents generally do not expect a strong effect. Respondents are divided on the extent to which the use of OTC products should depend on a doctor’s recommendation.

**Table 2:** Statements about over-the-counter drugs (*N* = 900). Question: *Which of the following statements about over-the-counter drugs do you agree with?*

Over-the-counter drugs	Mostly agree (in %)	Mostly disagree (in %)	Not specified (in %)
… are harmless	42	53	5
… have only minimal side effects	37	58	5
… can readily be taken as required	42	54	4
… should be taken only after a doctor’s recommendation	47	49	4
… are gentle, well tolerated	44	50	6
… are easy to use	62	33	5
… can also readily be taken by children	24	70	6
… have strong effects	23	73	4
… are very good for getting back into the swing of things	41	53	6
… often have a performance-enhancing effect	31	63	6
… can also be taken on a long-term basis	25	69	6
… can cause damage if taken incorrectly	63	31	6

Across all age groups, men in the sample were noticeably more likely than women to report that OTC products were harmless (52% vs. 31%, *p* < 0.001), had few side effects (48% vs. 26%, *p* < 0.001), could readily be taken as needed (52% vs. 31%, *p* < 0.001), were well tolerated (62% vs. 25%, *p* < 0.001), and were good for building yourself up or for convalescence (62% vs. 20%, *p* < 0.001).

## Discussion

### Main findings and interpretation

Over-the-counter drugs have become an indispensable part of medical treatment prescribed by GPs as well as of home management by self-medication. This is confirmed by previous studies showing high and regular use of OTC drugs [7, 8, 10, 21]. Like other studies, the results show that among over-the-counter products, pain-relieving drugs are particularly widely used [1, 18, 21].

The survey confirmed the finding of existing studies that women are more likely to consult doctors and pharmacists as well as the package information leaflet prior to using OTC preparations, to find out about risks and side effects. On the one hand, women seem to be better informed and more likely to have realistic expectations; on the other hand, they purchase OTC drugs more often [6, 7, 10]. This is confirmed by a large-scale online survey of patients in Germany dating from 2013 [20]. Here it was also found that products for dermal application or plant-based products were considered “rather not risky” among patients. As regards educational level, it is noted that members of a higher educational level purchase over-the-counter medicines more frequently than those of lower educational levels, partly due to their higher household income. This is confirmed, for example, by the work of Knopf and Mullan [13, 22]. In the present study, the financial income of the respondents was not determined, and no direct statements can be made about significant differences in terms of OTC consumption and attitudes in different financial situations. However, the study showed that respondents with different levels of education differ in terms of consumption and those with higher education are more likely to purchase OTC products. An analysis by Fereidouni et al. highlights that the experiences of self-medication could be classified into personal, social, organizational, and cultural categories [23].

Primary care patients associate OTC medications with the potential of gaining more personal responsibility for maintaining their health and often associate this group of medications with trivial illnesses, low dosages, and weak efficacy [2]. In Knopf et al. [7], a large majority of respondents also considered over-the-counter drugs to be suitable for treating complaints that do not justify a visit to the doctor.

Overall, the results suggest that a large proportion of patients are aware of the possible risks and side effects of over-the-counter drugs and therefore prefer, for example, to at least read the package information leaflet before taking them for the first time [24]. At the same time, the results show that there is a proportion of GP patients who potentially hold exaggerated or even erroneous attitudes towards OTC preparations and may therefore tend to underestimate their potential side effects. This is particularly true for men in the sample, who seem to be much more casual about using OTC drugs across all age groups. This often goes hand-in-hand with less willingness to consult a GP. In this context, a meta-analysis by Gualano et al. showed that 50% of adolescents use to take drugs without consulting a physician [25].

Undoubtedly, there is an advantage in the ready availability of OTC drugs, so that patients get their drugs in time when suffering from colds, sunburn, and headache. More dangerous might be the use of high-dose vitamins or hormones as often used as anabolics. For this reason, the strong presence of OTC drugs in advertising as a consumer influencer should not be underestimated. Advertising may disguise potential side effects and trivialize the use of the drugs in question [6]. Although it is challenging to conduct studies in this field and to clearly demonstrate advertising effects, several field experiments have been able to prove that attention and demand for medicines in general and OTC products in particular are induced by different advertising formats [26]. Against the background of his study, Block concludes that drug advertising can lead to increased health concerns and, thus, to an increased feeling of need, in particular due to the easy availability and applicability of OTC products [27]. An experimental study by Sauer comes to a similar conclusion [28]. According to Sauer, advertising does not only have the function of informing and motivating, but also a function of socialization. In view of the amount of advertising information received every day by recipients, this socializing effect can be very strong.

It is therefore all the more important that patients have a realistic idea of the capabilities and risks of OTC products. In addition to the advice provided by pharmacists, the trusting, long-standing support provided by GPs and their ongoing information and advice services play a central role in this [17, 24].

Overall, it appears to be of particular importance for GPs to regularly ask their patients about their consumption of over-the-counter drugs, consistently take account of polypharmacy and multimorbidity, and recommend OTC drugs accordingly [29, 30]. For example, in the context of multimodal pain management, appropriate therapeutic adjustment can prevent incorrect use of OTC analgesics and thus avoid adverse side effects or potentially harmful duplication of nonsteroidal anti-inflammatory drugs [31, 32]. In this way, it is possible to prevent drug therapy from aggravating the existing conditions and thus worsening the patient’s overall health status [33, 34]. Already established and proven instruments such as the Green Prescription for GP recommendation of pharmacy-only OTC preparations could be used more consistently in this context.

Last but not least, it is also essential that patients taking over-the-counter products are advised to report any health abnormalities promptly to their doctor [32]. Especially in a time of increasing self-medication, GPs have an indispensable role as a point of contact for providing guidance and ensuring patient safety [9].

In order to compare the results of the study better, Table 3 lists all researched studies in which the attitudes and consumption preferences of patients and consumers with regard to OTC drugs were determined in the course of a survey. Many respondents do not want to see a doctor over minor issues and think they can handle their health problems by self-medication. Some of them keep their OTC use from their treating physicians [1]. In several studies, the majority of respondents assume that they are able to self-medicate and to assess the advantages and risks of OTC products. Only some of the respondents rely on the leaflet. The comparatively high confidence in using OTC drugs correctly partly coincides with the finding that there are a not inconsiderable number of gaps in consumer knowledge, especially regarding the maximum daily dose, contraindications, and potential side effects. In most studies, the socioeconomic status or disposable income is not a significant influencing factor; in contrast, gender and age are important predictors with regard to attitudes and patterns of use of OTC products. In some studies, health awareness and health anxiety was a determining factor. This was not examined in the present study.

**Table 3:** Comparison of the study with available investigations (Population and patient surveys on OTC use)

Study	Eichenberg et al. [1]	Barrenberg et al. [2]	Knopf, Grams [7]	Beitz et al. [10]	Barrenberg, Garbe [20]	Mullan et al. [22]
Year of implementation	2012	2008–2011	2008–2011	1998	2013	2013/14
Type of survey	Paper-based survey	Computer-assisted personal interviews (CAPI), German Health Interview and Examination Survey for Adults	Computer-assisted personal interviews (CAPI), German Health Interview and Examination Survey for Adults	Face-to-face interviews, German National Health Interview and Examination Survey	Online survey, based on a quota sample with combined strata for age, gender, and education	Paper-based survey
Survey target group	Total population aged 14 and over, representative (Germany)	Total population aged 18–79, representative (Germany)	Total population aged 18–79, representative (Germany)	Total population aged 18–79, representative (Germany)	Adult participants (Germany)	Consumers, aged 18 years and over (Australia)
Sample size (*N*)	1976	7091	7091	7099	300	260
Key findings	Most common place to obtain information about OTC: pharmacies. Symptoms most commonly treated: cold symptoms, headaches. Most respondents do not want to see a doctor over minor issues. Good experiences with self-medication. Almost half of the sample keeps OTC use from treating physicians	Seven-day prevalence of OTC drug use significantly higher in women than in men. Female gender, an age of more than 60 years, reduced health status, drug use, and multi-morbidity identified as predictors of OTC drug use	Three quarters state they used at least one OTC preparation; prevalence highest among 70–79-year-olds. Overall, women have a significantly higher prevalence rate than men. Polypharmacy (use of 5 or more preparations) increases with age and is observed significantly more often in women. No differences in overall medication linked to social status	8% of men and 11% of women use self-medicated OTC drugs exclusively, whereas 12% and 29%, respectively, use OTC drugs in addition to prescribed drugs. Besides sex, factors such as age, education background, and community size determine self-medication. Most commonly used: vitamins, minerals, analgesics; most commonly reported indication for self-medication: “prevention.” Health consciousness appears to be a significant factor to explain the prevalence of OTC drug use	Seven-day prevalences of OTC drug use significantly higher in women than in men. Risk perception of specific OTC drugs impacted by the route of administration, the indication, and the drugs’ ingredients. Products for dermal application or plant-based products were considered “not risky.” 45% of consumers reported not reading the package leaflet of OTC drugs. People 60 years and older reported significantly lower levels of OTC drug off-label use than younger people	Knowledge of correct use of widespread OTC products was determined. A majority correctly identified ibuprofen as an active ingredient; a third couldn’t correctly identify the maximum daily dose and were unaware of some contraindications; 50% recognized potential side effects. Those who hadn’t completed high school were significantly less likely to seek medical advice and significantly less likely to know when it was safe to take these products

*OTC* over-the-counter

Overall, it would be advisable to give more attention to this public health concern and to promote initiatives (mass media campaigns, governmental actions) in order to make patients more aware of the risks with regard to consumption of drugs without medical consultation. Further studies on adverse effects are urgently needed.

### Strengths and limitations

The study investigates and describes a less-known topic.

Due to the limited case numbers and the regional recruitment focus, the study cannot claim to be representative. Moreover, one cannot rule out that medical practices that are interested in this subject signaled a greater willingness to have a waiting room survey conducted among their patients; the same applies to the willingness of the patients themselves to participate.

It is important to point out that this study was generally about over-the-counter medicines and their image or perceived potential among patients, not about specific groups of drugs, products, or circumstances of use. It will be the function of follow-up studies to shed a more nuanced light on this. In addition, future studies should include the views and experiences of doctors and pharmacists. To date, only a few papers have been published on this subject [35].

## Conclusion

Self-medication use among adolescents is a widespread phenomenon. The waiting room survey showed that over-the-counter drugs are widely used by primary care patients. OTC preparations are linked to a specific application profile, where priority is given to the treatment of cold symptoms, flu symptoms, and pain management. The common perception of over-the-counter drugs as simple-to-use, low-dose, and low-efficacy products does not necessarily correspond to the actual capabilities and risks of OTC drugs for self-medication. In particular, some of the men in the sample show an increased willingness to use the product and tend to underestimate potential side-effects. In light of these results, the provision of advice by doctors and pharmacists is of great importance.

### Supplementary Information

Appendix 1: Questionnaire
