# Supplementary material for: Attitudes, attributions, and usage patterns of primary care patients with regard to over-the-counter drugs—a survey in Germany
Source: Wien Med Wochenschr. 2022 Sep 23;174(3-4):61–8. doi: 10.1007/s10354-022-00967-6 (PMC10896805; doi:10.1007/s10354-022-00967-6)
Supplement: Supplementary file 1 — Appendix 1: Questionnaire [file 10354_2022_967_MOESM1_ESM.docx]

**1. Suppose you need a medicine that can be bought without a prescription: Do you still go to a doctor for advice beforehand?**


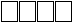
 **Yes, (almost) always**


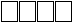
 **Yes, occasionally**


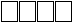
 **Yes, rarely**


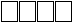
 **No, never**


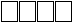
 **Not specified**

**2. How frequently do you to ask your doctor about the risks and side-effects of over-the-counter drugs?**


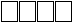
 **Frequently**


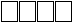
 **Occasionally**


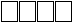
 **Rarely**


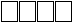
 **Never**


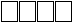
 **Not specified**

**3. How frequently do you ask your pharmacist about the risks and side-effects of over-the-counter drugs?**


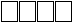
 **Frequently**


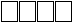
 **Occasionally**


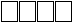
 **Rarely**


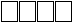
 **Never**


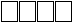
 **Not specified**

**4. How often do you buy over-the-counter drugs?**


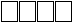
 **Frequently**


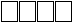
 **Occasionally**


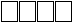
 **Rarely**


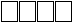
 **Never**

**5. Where do you usually buy over-the-counter drugs? (Multiple answers possible)**


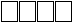
 **Pharmacy**


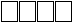
 **Online pharmacy**


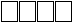
 **Other online mail order business (e.g. Amazon)**


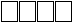
 **Drugstores and similar shops**


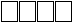
 **Supermarket**


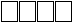
 **Other, namely: ________________________________**


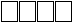
 **Not specified**

**6.** **While some over-the-counter drugs can be found in the health section of the supermarket, others can only be purchased at the pharmacy.**

**Do you think there are differences between these two types of over-the-counter drugs in terms of how they work and their strength?**


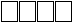
 **Yes, big differences**


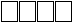
 **Yes, slight differences**


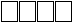
 **No, no differences**


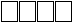
 **Not specified**

***Please only answer the following question if you also buy over-the-counter drugs on the Internet. If you do not, go directly to question 8.***

**7. Why do you buy over-the-counter drugs on the Internet? (Multiple answers possible)**


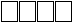
 **I save myself a trip to the pharmacy or shops.**


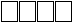
 **There is a greater choice of different preparations online.**


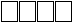
 **Online prices are cheaper than in pharmacies or shops.**


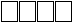
 **I prefer the anonymity of the Internet when shopping for drugs.**


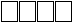
 **Other, namely: ________________________________**

**8. In your opinion or experience, for which complaints do you think over-the-counter drugs are particularly suitable? (Multiple answers possible)**


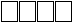
 **Colds/flu
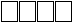
 Tension/stress**


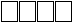
 **Headaches
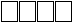
 Eye problems**


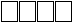
 **Insomnia
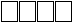
 Exhaustion/fatigue**


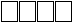
 **Low mood/depression
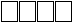
 Digestive problems/stomachache**


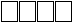
 **Bronchitis
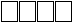
 Sunburn,** **insect bites**


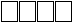
 **Skin problems/foot or nail fungus
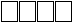
 Menopausal symptoms**


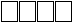
 **Prostate problems
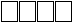
 Cystitis**


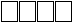
 **For prevention/prophylaxis
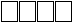
 Recovering after illness**


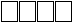
 **Other, namely: ________________________________**

**9. Here are several assumptions about over-the-counter drugs.**

**Which statements do you think are true and which are not?**

| **Over-the-counter drugs ...** | Completely true | Mostly true | Mostly untrue | Completely untrue |
| --- | --- | --- | --- | --- |
| **... are there to relieve the financial burden on health insurers, because patients have to pay for such drugs themselves** |  |  |  |  |
| **... have fewer side-effects than prescription drugs** |  |  |  |  |
| **... are not paid for by health insurers because there is no clear evidence of their efficacy** |  |  |  |  |
| **... are much easier to use and take than prescription drugs** |  |  |  |  |
| **... contain lower doses than prescription drugs** |  |  |  |  |
| **... save me from having to go to the doctor for every minor complaint** |  |  |  |  |
| **... are freely available to buy, because they have been on the market for a long time and are therefore well tried and tested** |  |  |  |  |

**10. Do you usually read the package information leaflet before using an over-the-counter drug?**


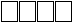
 **Mostly**


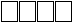
 **Rarely**


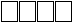
 **Never**


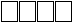
 **Not specified**

**11. Which of the following statements about over-the-counter drugs do you agree with?**

| **Over-the-counter drugs ...** | Completely agree | Mostly agree | Mostly disagree | Completely disagree |
| --- | --- | --- | --- | --- |
| **... are harmless** |  |  |  |  |
| **... have only minimal side-effects** |  |  |  |  |
| **... can readily be taken as needed** |  |  |  |  |
| **... should be used only after a doctor's recommendation** |  |  |  |  |
| **... are gentle, well tolerated** |  |  |  |  |
| **... are easy to use** |  |  |  |  |
| **... can also readily be taken by children** |  |  |  |  |
| **... have strong effects** |  |  |  |  |
| ... **are very good for getting back into the swing of things** |  |  |  |  |
| **... often have a performance-enhancing effect** |  |  |  |  |
| **... can also be taken on a long-term basis** |  |  |  |  |
| **... can cause damage if taken incorrectly** |  |  |  |  |

***Please only answer the following questions if you also obtain information about drugs on the Internet. If this does not apply, please go to the last page.***

**12. How often do you find out about drugs on the Internet, e.g. about areas of application or advantages and disadvantages?**


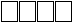
 **Frequently**


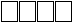
 **Occasionally**


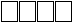
 **Rarely**


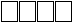
 **Never**


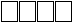
 **Not specified**

**13. Do you only look on the Internet for information about prescription drugs or about over-the-counter drugs as well? (Multiple answers possible)**


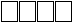
 **Prescription drugs**


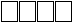
 **Over-the-counter drugs**


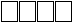
 **Both**


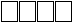
 **Not specified**

**14. Where do you get information about drugs on the Internet? (Multiple answers possible)**


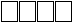
 **Manufacturer's website**


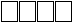
 **Health portals (e.g. Netdoktor, Jameda, Onmeda)**


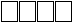
 **Consumer services websites (e.g. Consumer Advice Centre)**


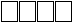
 **Websites of health guides and magazines (e.g. Apotheken-Umschau)**


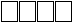
 **Health insurance company websites**


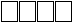
 **Other, namely: ________________________________**

*Finally, we need some statistical information from you. As with the rest of the questionnaire, this information will, of course, be treated in the strictest confidence and anonymized.*

**Your gender:**
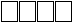
 **Male**
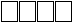
 **Female**
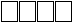
 **Other**

**Your age:** ______

**German State:**
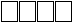
 Hesse
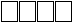
 North Rhine-Westphalia
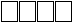
 Rhineland-Palatinate

**What is your highest educational attainment?**


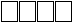
 **Primary/lower secondary diploma
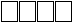
 Intermediate/high school diploma**


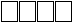
 **School leaving/vocational diploma
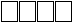
 (Technical) university degree**


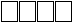
 **Other educational qualification
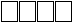
 No qualification**


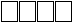
 **Not specified**

**Are you currently employed?**


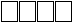
 **Yes
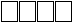
 No, pupil/student**


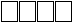
 **No, looking for work
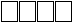
 No, other**


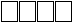
 **Pensioner**

**THANK YOU FOR TAKING PART!**
